# Current Status of Cherenkov-Based Gamma Detectors for TOF-PET and Proton Range Verification

**DOI:** 10.1109/trpms.2025.3579673

**Published:** 2025-06-13

**Authors:** Gerard Ariño-Estrada, Nicolaus Kratochwil, Stefan Gundacker, Emilie Roncali

**Affiliations:** Department of Biomedical Engineering, University of California at Davis, Davis, CA 95616 USA, and also with the Institut de Física d’Altes Energies, Barcelona Institute of Science and Technology, 08193 Barcelona, Spain.; Department of Biomedical Engineering, University of California at Davis, Davis, CA 95616 USA.; Institute of High Energy Physics, Austrian Academy of Sciences, 1050 Vienna, Austria.; Department of Biomedical Engineering, University of California at Davis, Davis, CA 95616 USA.

**Keywords:** Cherenkov radiation, gamma-ray detectors, medical imaging instrumentation, photodetectors, proton range verification (PRV), scintillation detectors, semiconductor detectors, time-of-flight positron emission tomography (TOF-PET)

## Abstract

Time-of-flight positron emission tomography (TOF-PET) and proton range verification (PRV) in proton therapy are based on the detection of gamma photons. Despite the difference in the ultimate goal and status of each of these two modalities, both heavily rely on the gamma detectors used in associated imaging systems. The emission of Cherenkov light has been studied extensively over the last decade as a gamma-detection signature in different detector configurations for TOF-PET and PRV. This review aims at: 1) capturing the breadth of works that report on using Cherenkov light for these applications from a detector instrumentation perspective and 2) summarizing barriers encountered by these approaches in their path toward commercial adoption. This review is structured in seven sections: I) brief introduction of TOF-PET and PRV needs that might be addressed with Cherenkov-based gamma detectors; II) physics of Cherenkov emission, propagation, and detection; experimental efforts in detector characterization grouped by the nature of the signals involved in the detector, i.e., III) simultaneous emission of Cherenkov and scintillation light; IV) pure Cherenkov emitters; and V) semiconductor detectors with simultaneous Cherenkov emission; Section VI consolidates the information with a special attention to challenges and potential strategies to overcome them; and Section VII concludes with a short paragraph. We hope this comprehensive review of the extensive work of researchers in this field in the last decade triggers further discussion and sparks inspiration among the community.

## I. Introduction

Time-of-flight positron emission tomography (TOF-PET) is instrumental in the detection of cancer, as well as other disorders, such as neurological and cardiological diseases. The performance of gamma detectors used in TOF-PET scanners, in particular the time resolution, is a key factor to improve the signal-to-noise ratio (SNR), and consequently many efforts in detector instrumentation in TOF-PET over the last decade have gone in that direction [1], [2], [3], [4].

Proton therapy is an advanced treatment method that uses the well-defined range of protons to precisely deliver the radiation dose to the tumor, minimizing the damage to the healthy surrounding tissue. The proton range must be verified down to the millimeter scale to fully benefit from this technique, ideally in real-time. However, proton range verification (PRV) in proton therapy is to-date an unmet clinical need. Online monitoring of the proton range during treatment would provide an added degree of certainty in the dose delivery and would help to further reduce safety margins to make proton radiotherapy (PR) more effective.

PRV using the prompt-gamma signature, in the form of prompt gamma imaging (PGI), has been the most studied modality [5], [6], [7], [8], although the use of other secondary radiation, such as proton-activated positron emitters, has been considered [9]. Different approaches have been proposed for PGI. Prompt gamma timing (PGT) uses the combined time-of-flight of the proton and the prompt-gamma to infer shifts of the proton range in the direction of the proton beam. Similarly to TOF-PET, the time resolution of the gamma-detectors is crucial in this modality.

Cherenkov light has been studied over the last decade as a mechanism for gamma-ray detection for TOF-PET motivated by its potential to provide an accurate detection time. More recently, Cherenkov light has also been studied for PRV via PGT with similar motivations.

The studies related to Cherenkov light for TOF-PET and PRV detectors comprise simulation studies of the generation and detection of Cherenkov light, measurements with small crystals of different materials, and different readout electronic strategies. Such research has been almost entirely at the benchtop level and no system-level setups have been reported to-date.

In this review, we aim to highlight the most important outcomes of the research related to Cherenkov light for gamma-detection and discuss potential follow-up research based on the state-of-the-art technology. We also emphasize key steps needed to continue moving the field toward full-systems, based on such approach.

Developments in TOF-PET and PRV achieved with other kinds of detectors, such as scintillation-based detectors, were not included in this review unless concepts have importance in the Research and Development of Cherenkov-based gamma detectors. Notwithstanding their relevance and potential impact in the field, our goal is to focus on the Cherenkov emission process as a gamma detection signature for such medical applications.

We have structured the body of the article in seven sections. The first section introduces the motivation and context for using Cherenkov-based gamma detectors in TOF-PET and PRV. In the second section, we review the physical foundation of Cherenkov light emission as well as its modeling with simulation tools. The following three sections capture the developments in radiation detection instrumentation grouped by the use of Cherenkov light: section three covers detectors with simultaneous Cherenkov and scintillation light, section four covers those where Cherenkov is the only detection mechanism, and section five includes semiconductor detectors that also emit Cherenkov light. We further devote one section to discuss different research avenues that, based on the achievements reported in the literature, can advance the use of Cherenkov light as a gamma detection principle in full-scale systems for TOF-PET and PRV. In section seven we conclude the article.

## II. Physics and Modeling of Cherenkov Light

In 1889, Oliver Heaviside predicted for a charged particle traveling at a speed greater than the speed of light in a medium that “The displacement cannot be outside a certain cone of semi-vertical angle whose sine equals the ratio v/u of the speed of light to that of the charge” and that “[the displacement] must form [⋯] an electromagnetic wave” [10]. Independently, Arnold Sommerfeld also predicted the emission of such electromagnetic wave in 1904 [11].

From Marie Curie’s biography, published in 1937, it reads that she attributed the glow of light in solutions with radioactive emitters to fluorescence in 1910: “Radium had something better than “a beautiful color”: it was spontaneously luminous: [⋯] their [radium-rich particles] phosphorescent bluish outlines gleamed, suspended in the night” [12]. First evidences of a specific light emission were reported by Leon Mallet between 1926 and 1929 [13], [14], [15].

Pavel Čerenkov published a substantial study on this phenomenon in 1934 [16] and 1937 [17] that served to name the phenomenon after him. Frank and Tamm developed a theory that explained Cherenkov light from a classical electromagnetism perspective [18], [19]. Jelley [20] provided during the 1950s an overview of the physics of Cherenkov radiation as well as the experiments carried out during the 1930s to study the effect. These efforts constitute most of the Cherenkov emission theory we have to-date.

High-performance modeling of Cherenkov light aimed at preclinical nuclear imaging was studied by Mitchell et al. [21] in 2011. In that study, Cherenkov light was generated in the organic tissue of the animal by the positron emitted by the radiotracer and was detected with an optical imaging system. Numerous publications on modeling and experiments followed and constituted an important foundation for the first efforts on the use of Cherenkov light as a mechanism for gamma-ray detection in medical imaging. It is worth noting that in the latter case, the particle emitting Cherenkov light is the electron generated by the Compton, photoelectric, or electron-positron pair creation interaction between the gamma-ray and the detection medium. Hereafter in this section, we focus on the modeling of its emission, propagation, and detection in separate subsections.

### A. Emission

Gamma-ray interactions can result in Cherenkov light emitted when the positrons or electrons are transferred a kinetic energy above the Cherenkov threshold in that medium. The Cherenkov threshold in one medium is determined by its index of refraction *n* following (1), where *c* is the speed of light and *m*_*e*_ is the mass of the positron and the electron. This threshold is material dependent and typically ranges between 50 and 180 keV kinetic energy

(1) $$E_{th}=m_{e}c^{2}\left(\frac{n}{\sqrt{n^{2}-1}}-1\right).$$

In TOF-PET these processes are photoelectric absorption and Compton scattering. In the first case, electrons in the inner shell of the atom are more likely to undergo this interaction, therefore the electron receives the gamma-ray energy minus the K-edge binding energy. In the second, part of the energy is transferred to the electron, which is often in the outer part of the shell. The transferred energy is defined by the Compton relation in (2), where *E*_0_ and *E*_*f*_ are the initial and final energy of the gamma, and *θ* is the scattering angle

(2) $$E_{f}=\frac{E_{0}}{1+\left(\frac{E_{0}}{m_{e}c^{2}}\right)(1-\text{cos}\theta )}.$$

In the PRV application, provided that prompt-gammas have energies well above 1.022 MeV, the pair creation mechanism adds to the photoelectric and Compton interactions. In this case, both the positron and electron receive energy from the prompt-gamma and do emit Cherenkov light while their kinetic energy is above the Cherenkov threshold in that medium.

The number of generated Cherenkov photons by an electron is described by (3), where *λ* is the wavelength of the emitted photons, *β* = *v/c* the speed of the electron, *α* the fine structure constant, *n* the refractive index and *z* = 1 the charge of the electron

(3) $$\frac{d^{2}N}{dx\;d\lambda }=\frac{2\pi \alpha z^{2}}{\lambda^{2}}\left(1-\frac{1}{\beta^{2}n^{2}(\lambda )}\right).$$

The predicted number of generated Cherenkov photons for 511 keV gamma depositions is up to 33 photons for different crystals [22], [23], [24], [25], [26] with several aspects affecting the result significantly. For example, the range of integration of *λ* can vary by a few tens of nanometers depending on where the cutoff value of the material is set. Another challenge is that often values for index of refraction in the ultraviolet range are not available and many arbitrary models are used. On the track of the electron, the *x* increments where the total number of generated Cherenkov photons is “updated” are also arbitrary and makes modeling challenging. Lastly, the binding energy of the electron, the refractive index, and the material density determine the range of the recoil electron where Cherenkov photons can be produced [27].

Table I shows a list of materials with their physical properties and a prediction of the number of Cherenkov photons generated using the same numeric implementation [28] of Frank–Tamm equation. The Cherenkov emission profile follows a 1/*λ*^2^ distribution with more photons emitted at lower wavelength [(3)]. This makes materials with high transparency in the UV and VUV more attractive, at the same time it poses challenges in the photon detection efficiency at such short wavelength.

**Fig. 1.**
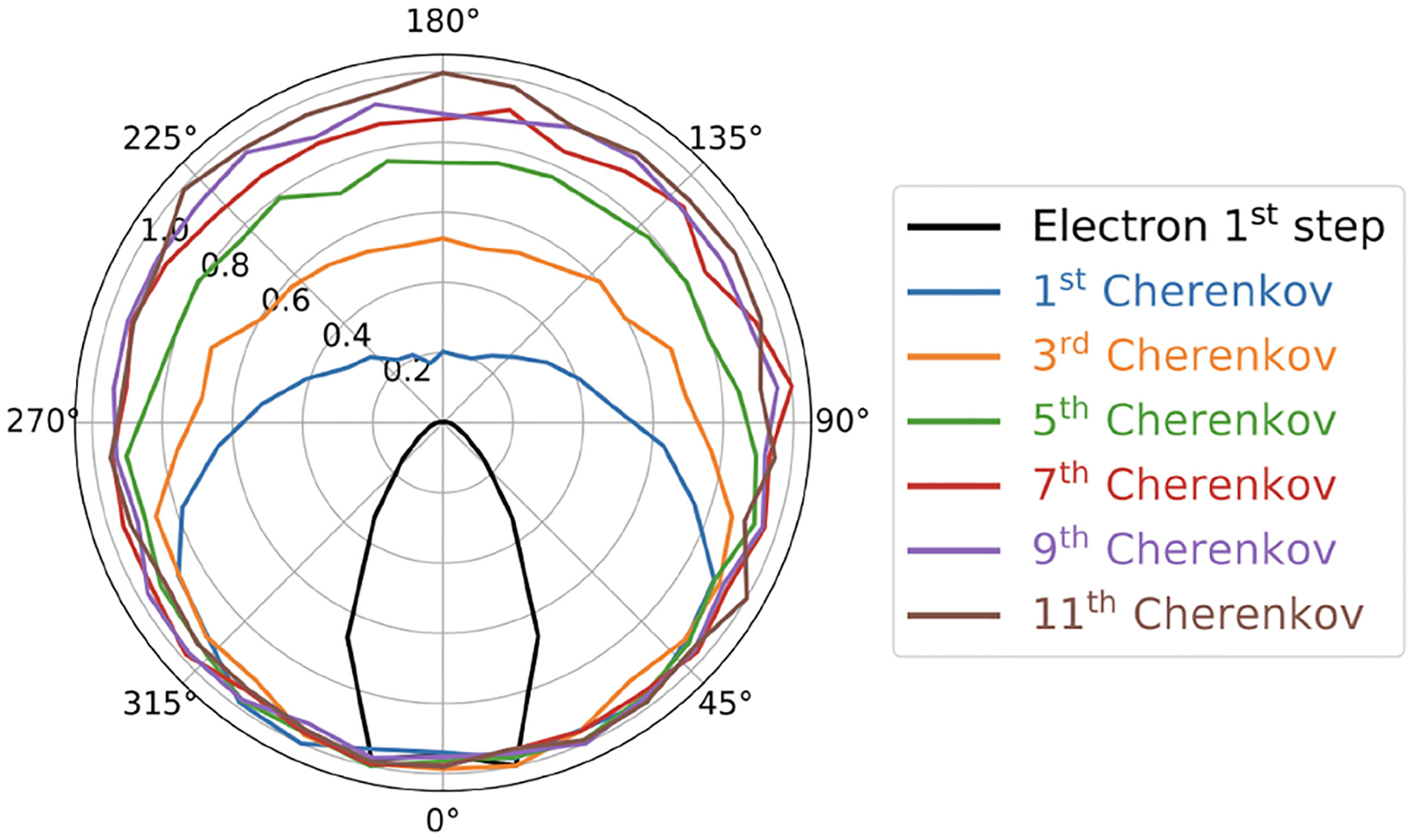
Directionality of the electron and some of the first Cherenkov photons emitted after the interaction of a 511 keV gamma with a BGO crystal. Courtesy of Dr. Carlotta Trigila.

Trigila et al. [29] showed that limiting the electron velocity change per step in the electron propagation in Geant4, and thus the total number of steps could change the predicted Cherenkov generation from 14 (fewer steps) to 17 photons (more steps). This study suggested that for an electron with an initial kinetic energy of 425 keV, a higher number of total steps (≈ 1800) and short mean step length (≈ 50 *μ*m) were modeling the number of emitted Cherenkov photons more accurately. This study also showed that the first emitted Cherenkov photons had a strong correlation with the initial electron direction, but this correlation weakens for the Cherenkov photons produced later (Fig. 1).

### B. Propagation

Some of the aforementioned works studied the correlation between the direction of the ejected electron and that of the generated Cherenkov photons, with mixed results, ranging from low to no correlation [22], [25], [29], [30]. In the absence of a robust demonstration of forward directionality, in this section we will assume most of Cherenkov light is emitted isotropically. For high aspect ratio crystals this means that half of the photons are propagating in the opposite direction of the incident gamma-ray.

The resulting two main waves of photon propagation (front and back) have been simulated in [31] and [32], or experimentally displayed in [30] and [33], and the impact on the time bias is described in depth in [34] (for scintillation light). In [32], the depth of interaction (DOI) contribution for a 20-mm long LSO-like crystal was estimated to be around 60 ps. Cramer–Rao lower bound calculations based on Monte Carlo generated arrival times for 2 × 2 × 20 mm^3^ and 3 × 3 × 20 mm^3^ LYSO crystals were performed in [31] and [35], respectively, extending the applicability of such calculations for non-negligible photon time spread. These calculations were extended toward purely mathematical expression on the light transport [36], removing the need for exhaustive Monte-Carlo simulations to determine the optical time spread. A first attempt to merge Cramer–Rao lower bound calculations with the detection of prompt photons was done in [23] by adding a Dirac-delta function at the start of the scintillation. However, neither the intrinsic DOI limitation nor event-to-event fluctuations on the number of detected prompt photons was included. The latter was done in [37], highlighting the importance of keeping population of photons separated and outlining timing improvements if the event-to-event fluctuations on the number of prompt photons can be effectively monitored [38].

### C. Extraction and Detection

Measurements with diverse Cherenkov emitters [PbF_2_, BGO, TlBr, thallium chloride (TlCl)] consistently report between 2 and 5 detected Cherenkov photons using SiPM with state-of-the-art quantum efficiency and slightly different readout electronics when detecting 511 keV gammas [26], [30], [33], [39], [40]. Several parameters influence the extraction of photons from the crystal toward the photodetector, such as the material at the interface, the difference in index of refraction of the crystal, the interface and the photodetector [41], or the surface treatment [42], [43] and optical reflectors [44], [45] of the crystal in all its sides.

**Fig. 2.**
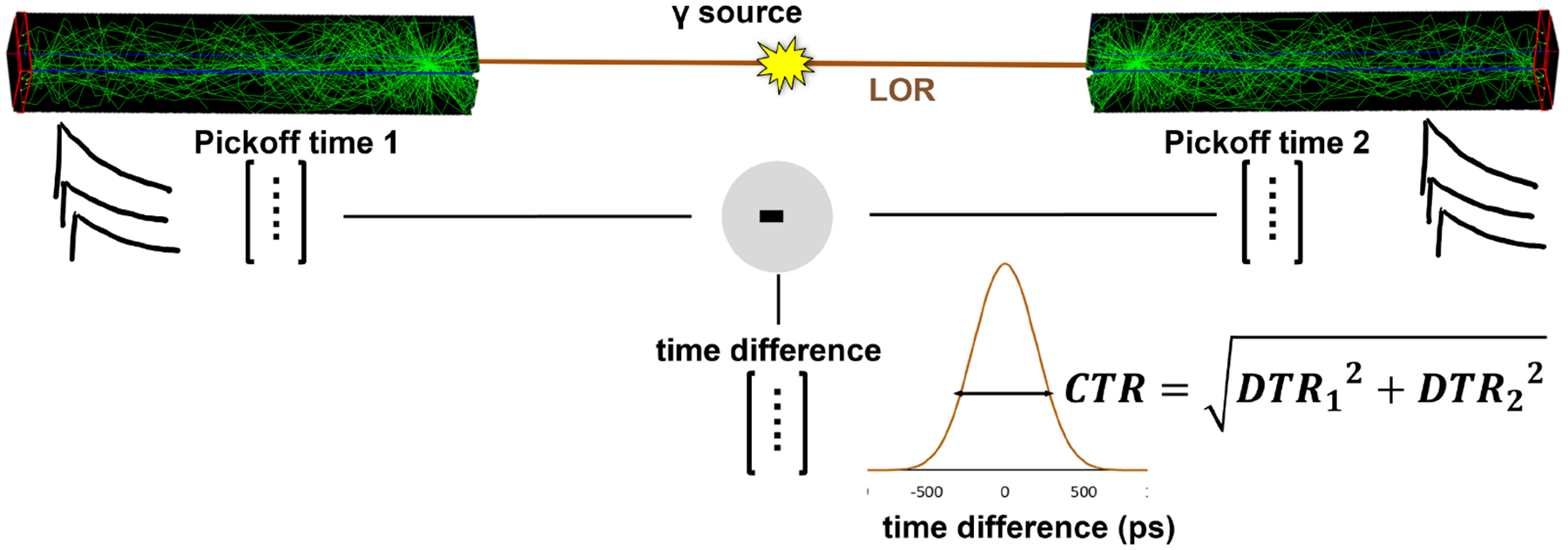
Illustration of a conventional geometrical model used to evaluate CTR performance between two crystals. (The CTR is the quadratic sum of the individual detector time resolutions - DTRs).

Stockhoff et al. [46] presented an optical model based on GEANT4 that accounts for all such factors. This model was used by Roncali et al. [25] to predict the influence of the aforementioned factors in the detection of Cherenkov light specifically. For a short BGO crystal wrapped with Teflon, an average of 3.4 Cherenkov photons are detected, which represents 18% of the emitted prompt photons. Similar fractions of detected Cherenkov photons were predicted and validated against experimental data with TlCl and TlBr with the same simulation framework [40]. The coincidence time resolution (CTR) of such crystal predicted by this model was validated against experimental data using a CTR measurement setup, as illustrated in Fig. 2

The same model was used by Rebolo et al. [47] to predict the emission of Cherenkov photons in a 1 cm^3^ crystal exposed to gamma-rays with 2.3, 4.4, and 6.1 MeV. The number of detected Cherenkov photons depended on the material (PbF_2_, TlBr, and TlCl) and photodetector choice and ranged between approximately 80 and 150.

## III. Scintillation and Cherenkov Detectors

In this section we include detectors based on materials that feature Cherenkov light but are dominated by the emission of scintillation light. Cherenkov and scintillation light can combine the high potential for time resolution of the first with the robust energy discrimination and reasonable spatial resolution of the latter.

This section has been divided in six subsections, starting with the first CTR measurements that intentionally used the Cherenkov component in BGO. Second, we capture the improvements in readout electronics, that contributed to further improved measurements. Third, we include data corrections used in BGO measurements to improve CTR. In the fourth section we aim to capture time improvements obtained with multiple photodetector readout in a single crystal. Fifth, we report on the improvements of SiPMs. Finally, we report on simultaneous Cherenkov and scintillation emitters other than BGO.

### A. First Measurements With BGO

BGO is the most studied scintillation and Cherenkov emitter in PET. BGO has a high refractive index and transmittance in the UV-range down to 310 nm, that make it an excellent Cherenkov emitter. Additionally, it has a higher detection efficiency and lower production cost compared to LSO, which make it an attractive material for this application.

**Fig. 3.**
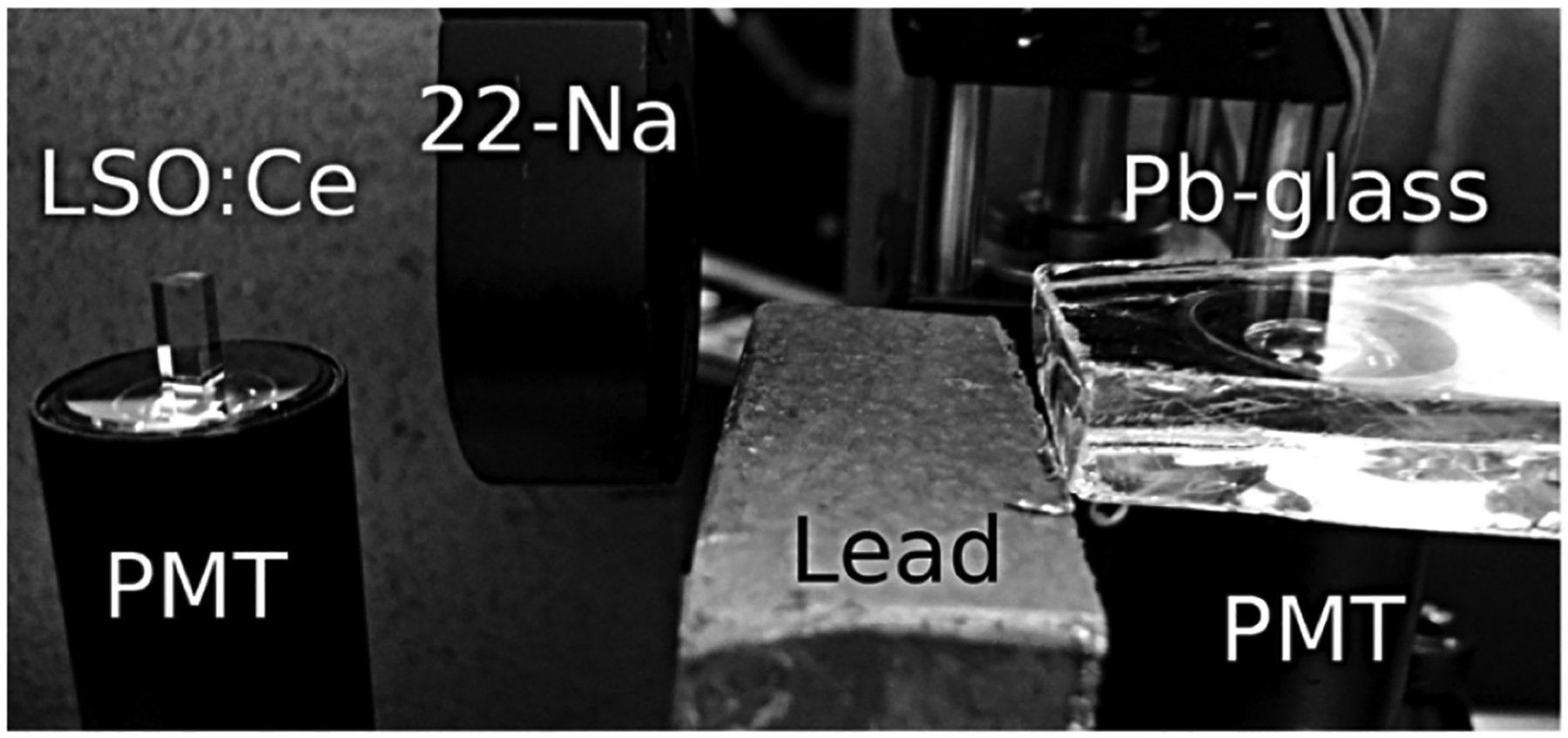
Photograph of the setup used to obtain the first CTR measurements. Adapted from [22].

Brunner et al. [22] were among the first researchers that investigated the presence of Cherenkov photons simultaneously with scintillation light. This study simulated the influence of prompt photons on the scintillation rise time and determined the Cherenkov photon yield for different materials. A proof-of-concept experiment was also conducted between an LSO reference detector and a Pb-glass (Fig. 3).

Kwon et al. [48] reported the first experimental study that purposely utilized both scintillation and Cherenkov light. It was conducted by coupling BGO crystals to analog SiPMs and measuring the CTR upon 511 keV photopeak selection. An important aspect of this study was to set the leading edge threshold (LED) below the signal amplitude of a single triggered cell, thus the timestamps are mostly generated by Cherenkov light. The resulting time delay distributions were fitted with Lorentz functions yielding CTR values between 267 ps FWHM (2 × 2 × 3 mm^3^) and 562 ps FWHM (3 × 3 × 20 mm^3^).

The following year, digital SiPMs together with BGO were used by Brunner and Schaart [24] to trigger more effectively on the first detected photon, thus reducing the contribution of the readout electronics. CTR values improved to 200 ps and 330 ps FWHM for 3 and 20-mm thick crystals, respectively.

The ratio of prompt to scintillation light for different materials was evaluated by means of time-correlated single photon counting measurements by Gundacker et al. [23]. This study also showed the importance of detecting prompt photons with good single photon time resolution (SPTR) and small photon time spread and simulated the achievable CTR as function of the number of prompt photons.

### B. Improvement of the Front-End Electronics

**Fig. 4.**
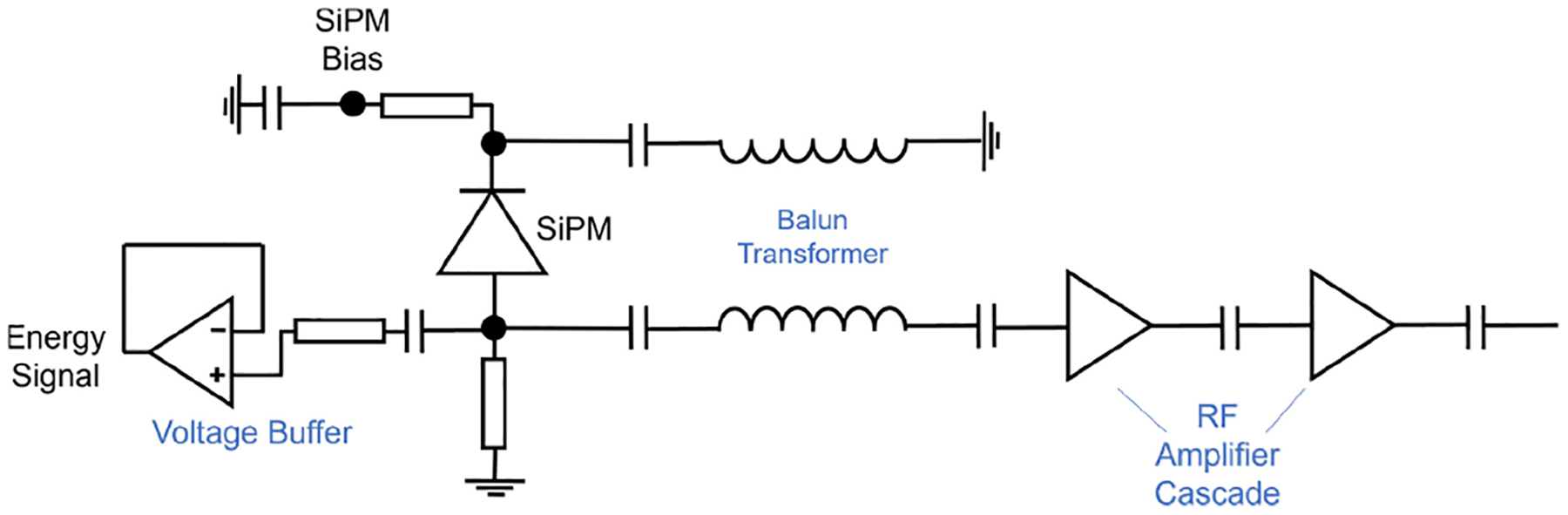
Schematic of the low-noise high-frequency readout. Courtesy of Dr. Josh Cates.

Front end readout electronics plays a critical role in achieving low SPTR of analog SiPMs and therefore to leverage the prompt photon signature. Cates et al. [49] used a passive compensation circuit where a balun transformer is connected between the SiPM anode and cathode in a balanced-to-unbalanced configuration to two radio-frequency amplifiers in cascade (Fig. 4).

This custom low-noise and high-frequency readout electronics yields a steep signal slew rate (dV/dt) and therefore minimizes the influence of electronic noise on the SPTR. SPTR values of less than 100 ps FWHM were measured even for 4 × 4 mm^2^-sized NUV-HD SiPMs from Fondazione Bruno Kessler (FBK), paving the way for CTR improvements with Cherenkov light.

In Gundacker et al. [50], the advancement of using such optimized readout electronics was shown by measuring the CTR of two small (2 × 2 × 3 mm^3^) BGO crystals coupled to FBK NUV-HD SiPMs. The time delay distribution was modeled with a sum of two Gaussian distributions yielding CTR values of 158 ps FWHM and 350 ps full width at tenth maximum (FWTM). The timing capability of different BGO crystal thicknesses and similar readout electronics was also evaluated by Cates and Levin [51] with CTR values between 200 ps (3 × 3 × 3 mm^3^) to 277 ps (3 × 3 × 15 mm^3^) FWHM.

### C. Data Corrections

With about 17–18 Cherenkov photons produced in BGO upon 511 keV gamma-ray deposition [26], only a fraction of them are detected and the number shows great fluctuations leading to time-walk effects. In Kratochwil et al. [38] these fluctuations were monitored by investigating the SiPM signal rising time, which allows to correct for the associated time walk effects. Additionally, events were classified in “fast” and “slow” categories on an event-to-event basis, improving the CTR as more Cherenkov photons were detected. For the fastest 20 % of the events per channel (4 % in coincidence), CTR values of 117 ps and 200 ps FWHM were measured for 2 × 2 × 3 mm^3^ and 2 × 2 × 20 mm^3^ BGO crystals coupled to NUV-HD SiPMs and the overall CTR values (all photopeak events) improved to 151 ps and 259 ps, respectively.

**Fig. 5.**
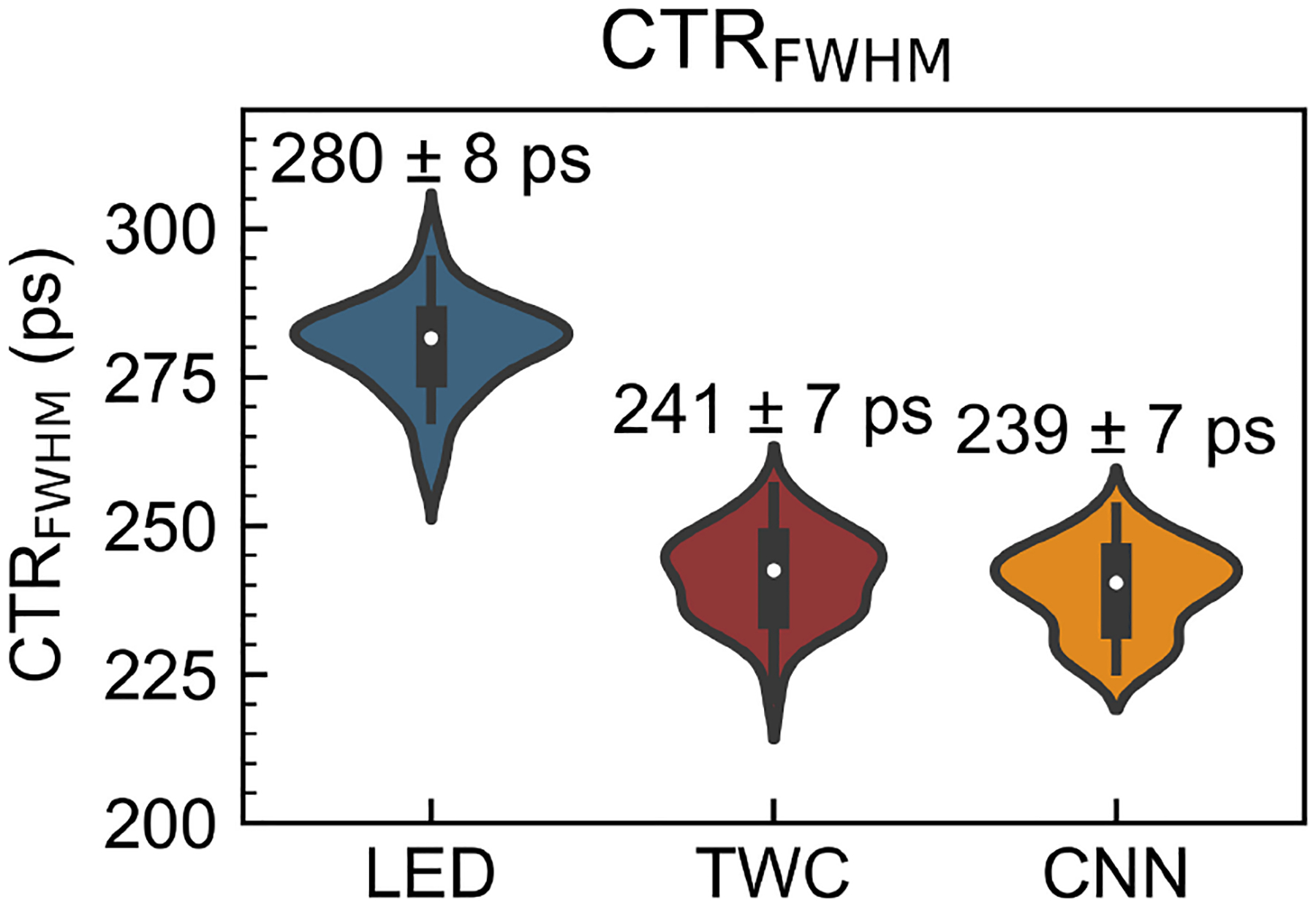
Violin plots showing the CTR results after correction for LED, TWC, and NN. Bootstrapping with 50 resampling was used to gauge the performance variability (larger width = more frequent) of the method. From [52].

Using a very similar acquisition setup, Loignon-Houle et al. [52] studied the potential of time walk correction (TWC) and convolutional neural networks (CNN) by using two timestamps corresponding to two thresholds applied to the rising edge of the SiPM signal. For two opposing BGO crystals of size 2 × 2 × 20 mm^3^, both TWC and CNN showed a comparable improvement of ≈15%, leading to a final result fo 240 ps FWHM. The CNN result, however, showed more consistent performance (Fig. 5) and a more effective tail reduction in terms of FWTM in the coincidence time distribution than TWC.

Following the idea on event classification, a semi-monolithic BGO photon counting detector was proposed by Cates et al. [53]. In a proof-of-concept study using high frequency readout electronics and 16 individual 2 × 2 mm^2^ NUV-MT SiPMs coupled to a 2 × 20 × 43 mm^3^ BGO plate, photons could be counted in an almost digital-like manner [54]. The extracted probability density function on an event-to-event basis paves the way for sophisticated data correction methods with estimated CTR values of 240 ps in coincidence.

### D. Dual-Ended BGO Configurations

Kwon et al. [55] studied the improvement by coupling one SiPM on each end of the crystal, dual-ended (also referred to as double-sided readout), versus the conventional, single-ended, readout strategy. The benefits of dual-ended vs single-ended are twofold: First, it increases the total active surface coupled to a photodetector, and second, adds an additional signal. The study proposes an adaptive time difference calculation, where either of the two SiPM signals is chosen based on a comparison criterion. With such an approach, a CTR of 331 ps FWHM was estimated for two 20 mm thick BGO crystals in coincidence.

Kratochwil et al. [56] measured the CTR in dual-ended BGO crystals with dimensions of 3 × 3 × 20 mm^3^ with low-noise high-frequency readout electronics [49] and applying an adaptive timestamp weighting. The best results for different categories of events ranged between 175 ps and 268 ps FWHM with an harmonic average (all photopeak events) of 234 ps.

The dark count and crosstalk contributions due to the interaction between opposed SiPMs was measured and modeled with this same setup [57]. The dual-ended readout configuration with the two SiPMs biased showed up to 50% higher dark count rate and more than twofold increased crosstalk probability at high overvoltage, compared to the single-ended case.

He et al. [58] studied the potential mitigation of the light propagation contribution to the total time-jitter by using dual-ended photodetector readout. Simulations predict this contribution can be reduced to a range of 20 ps to 50 ps depending on material, crystal length, and DOI resolution.

### E. SiPM Improvements

**Fig. 6.**
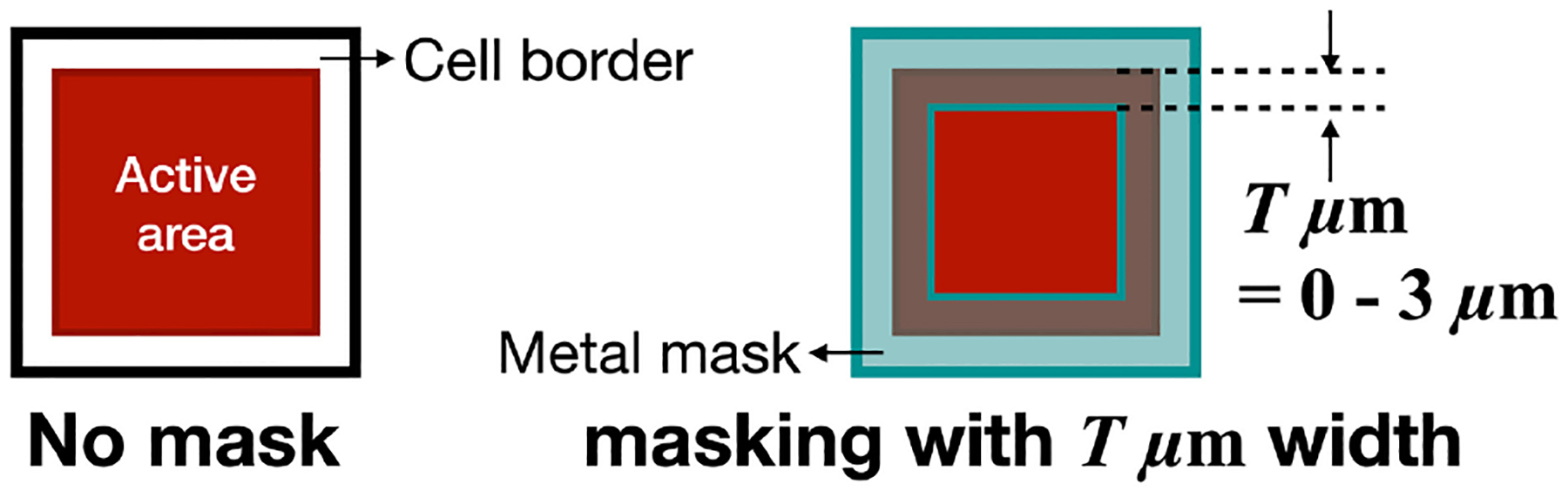
Illustration of the SiPM masked used in [59]. Courtesy of Dr. Sun Il Kwon.

Improvement of SPTR in SiPMs benefits the extraction of the fast component of Cherenkov light. Specific directions have been taken to exploit the prompt nature of Cherenkov light in BGO using SiPMs. Gundacker et al. [59] presented an SiPM design with a mask covering the borders of the microcell (Fig. 6).

Devices with the mask showed an improved SPTR, attributed to a better extraction of the fast components of the signal. An SPTR of 28 ps FWHM was reported for an SiPM with 1 mm^2^ active area and a 3 *μ*m mask on microcells.

Next, Cherenkov radiators benefit from improvements in photon detection efficiency- especially in the NUV and VUV- and reduction of correlated SiPM noise. Noteworthy are the efforts by FBK and Broadcom with metal-filled trenches between the SiPM cells (NUV-MT), which significantly reduces the SiPM crosstalk and thus allows to operate the devices at higher excess voltage [60]. Nadig et al. [61], Piller et al. [62], and Lee et al. [63] report CTR values for small BGO crystals of 109 ps, 123 ps, and 111 ps FWHM, respectively, obtained using high frequency readout and biasing the NUV-MT SiPMs at up to 20 V above the breakdown voltage.

Another emerging pathway, combining the flexibility of analog SiPMs and the high performance of digital SiPMs, is a hybrid detection design [64]: Multiple SiPM cells are combined into one *μ*SiPM [65], and the signal of all the *μ*SiPMs is captured, instead of only one from the unsegmented SiPM. Advantages of segmented SiPMs are better SPTR (better intrinsic SPTR and lower capacitance, therefore steeper signal) [66], both of great advantage for Cherenkov photons. In addition, multiple (digital-like) timestamps can be obtained per gamma-ray interaction with associated data corrections outlined in Section III-C and presented in Yi et al. [67] with two mini SiPMs (1.4 × 2.5 mm^2^).

### F. Other Cherenkov and Scintillation Emitters

While the majority of research and development is based on BGO, it is not the only material with a strong Cherenkov signature on top of the scintillation emission (see Table I).

Bismuth silicate (BSO) exhibits similar crystal properties compared to BGO, with the main difference of a lower scintillation light yield (≈ 2 ph/keV) and a faster scintillation decay time [68]. Silicon and germanium can be mixed (Bi_4_(Ge_*x*_Si_1−*x*_)_3_O_12_) toward a tunable scintillation emission between pure BSO and pure BGO, while the Cherenkov profile is mostly unaffected [69].

**Fig. 7.**
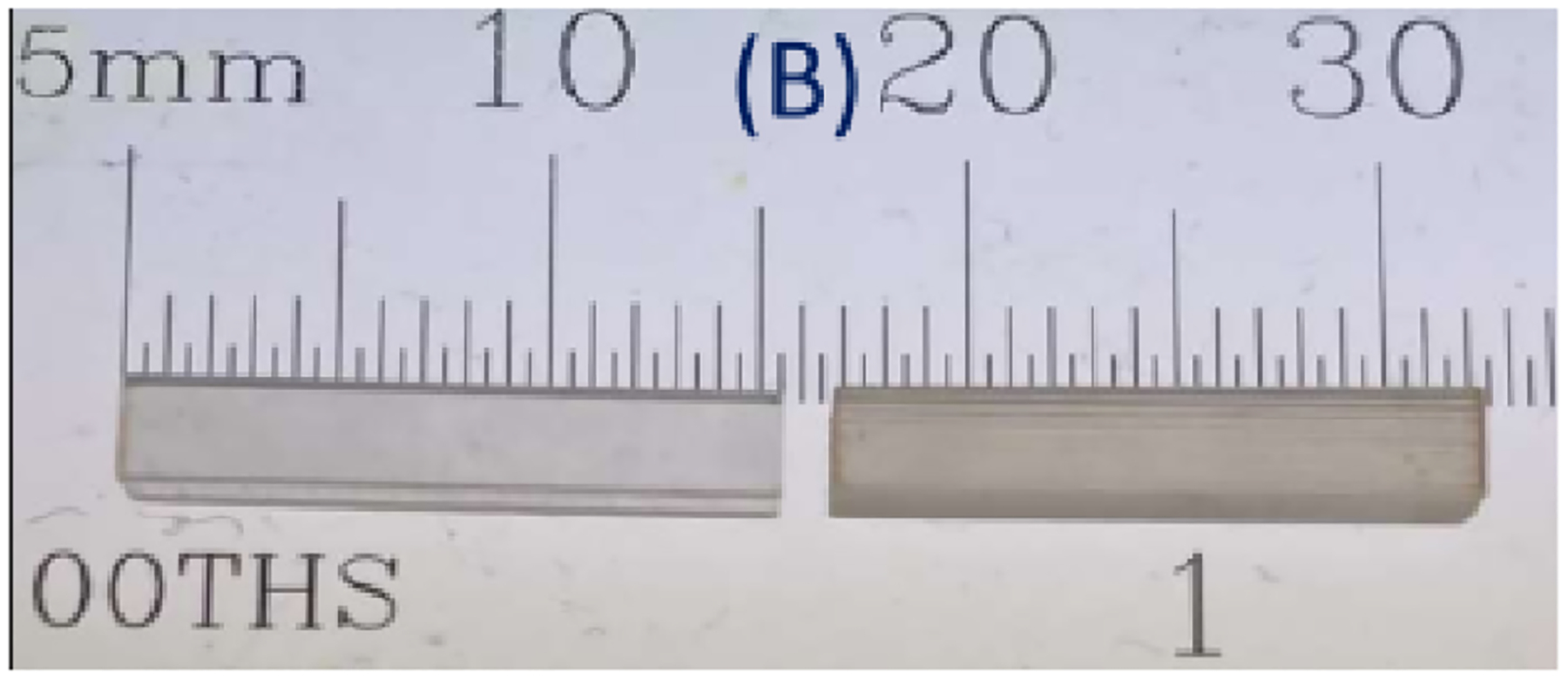
TlCl crystals. Adapted from [28].

Pure TlCl does not scintillate and with the Cherenkov signature only, CTR values (against a reference detector) between 300 to 400 ps were reported by Ariño-Estrada et al. [40]. These values could be improved to 210 ps by Terragni et al. [33] by employing high-frequency readout and a TWC. By doping TlCl with beryllium and iodine (TlCl:Be,I) [28], it becomes a scintillator with multiple decay times and a light yield of about 0.9 ph/keV. The CTRs with TlCl:Be,I crystals upon 511 keV photopeak discrimination with dimensions of 2.8 × 2.8 × 2.8 mm^3^ and 2.8 × 2.8 × 15.2 mm^3^ were 235 and 402 ps FWHM, respectively (Fig. 7). It is worth noting that the effective atomic number and photofraction of TlCl to 511 keV gammas is 76 and 46%, respectively, both greater than BGO.

In the same manner, certain crystals only show a pronounced scintillation component in addition to a possible exciton emission upon doping (e.g., GAGG, LSO, LuAG, LYSO, YAG). Carefully changing doping and co-doping concentrations might be a pathway to engineer the scintillation emission in terms of light yield and decay times, and thus the density ratio between prompt and slow emission. However, in many instances the scintillation dominates and, although present, the faint Cherenkov signature cannot be detected and does not play any relevant role in the Research and Development [23].

Lead tungstate (PbWO) is discussed briefly in Section IV with sole Cherenkov radiators as it has a very faint but fast (≈ 10 ns) scintillation emission of around 0.02 ph/keV. The number of scintillation and Cherenkov photons is very similar, hence, it does not fully benefit from energy discrimination capabilities [70]. Research and Development efforts with PbWO are therefore covered in the following section.

## IV. Sole Cherenkov Detectors

In this section we refer to pure Cherenkov emitters as crystals in which Cherenkov light is the dominant (i.e., limited to no scintillation) over the scintillation process. Pure Cherenkov emitters provide exciting features and challenges in equal amounts. On the one hand, the prompt and neat emission of Cherenkov light has the potential to result in fast gamma detections with high SNR. On the other hand, the much lower light intensity compared to scintillation light tends to yield a lower detection efficiency for TOF-PET systems. An additional limitation of this kind of detectors is the loss of proportionality between the detection of optical photons and the deposition of energy in the crystal compared to the one obtained with scintillation light. Lastly, correlated and uncorrelated photodetector noise is indistinguishable from low intensity true signals adding difficulties when operating at ambient temperature.

This section is divided in three parts: First, a chronologic layout of the efforts in ultrafast timing, which have obtained the most competitive time resolutions among the TOF-PET instrumentation community. Second, an overview of timing measurements with SiPMs and Cherenkov radiators with emphasis on both high sensitivity and fast timing. And third, the advances in pure Cherenkov emitters in PRV via PGI.

### A. Ultrafast Measurements With MCP-PMTs

The use of pure Cherenkov emitters for TOF-PET was initially proposed by Ooba et al. [71] and Miyata et al. [72] in the mid 2000s. The latter reported a CTR of 170 ps FWHM with two lead glass crystals with 10-mm thickness coupled to microchannel plate photomultipliers (MCP-PMT) and read out by analog electronics. Dolenec et al. [73] used the same photodetectors to obtain CTR values of 160 ps (68 ps *σ*) and 184 ps FWHM (78 ps *σ*) for black-painted PbF_2_ crystals with 5 and 15 mm thickness, respectively. The same group showed how cross-talk [74] suppression in the photodetector improved CTRs for the black painted PbF_2_ crystals with the same dimensions to 71 ps FWHM (equivalent to 5 mm) and 87 ps FWHM (37 ps *σ*) (equivalent to 15 mm) [75]. Reported in the same study, results for PbWO_4_ crystals with the same paint and dimensions deteriorated between 15% and 35%.

**Fig. 8.**
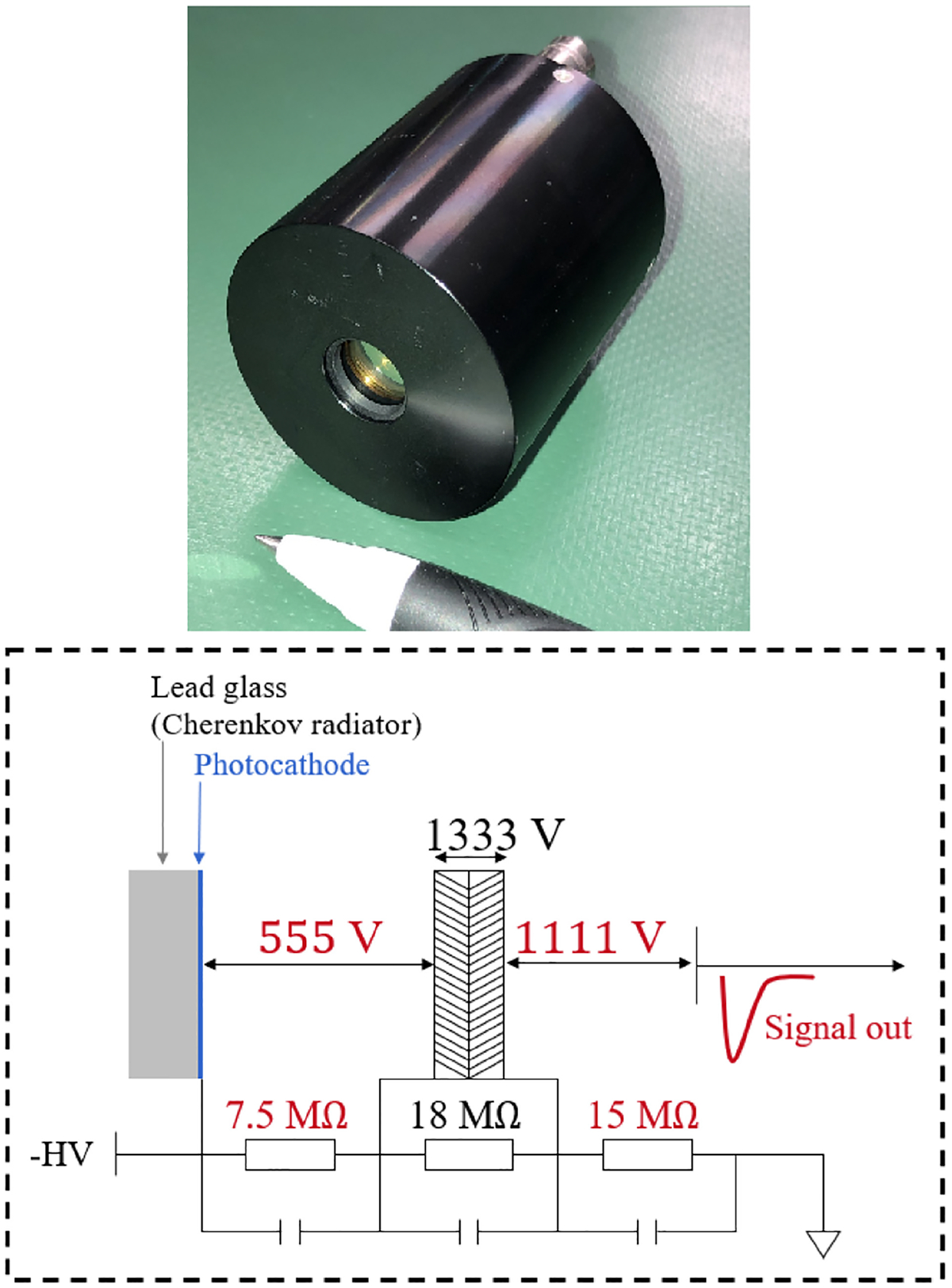
Top) Picture of the MCP-PMT. Bottom) Diagram of the MCP-PMT. Courtesy of Dr. Ryosuke Ota.

In Ota et al. [76] single-channel MCP-PMTs, depicted in the top of Fig. 8-top, yielded a CTR of 46.9 ps FWHM coupled to PbF_2_ crystals with 5-mm thickness, wrapped with black tape, and with different cross sections.

The publication attributed the improvement compared to the measurements in [75] to a significant improvement of the SPTR of the MCP-PMTs, which was estimated to be 25 ps. Ota et al. [77] introduced a 3-mm layer of lead glass inside the same device (bottom of Fig 8), hereafter referred to as CRI-MCP-PMT, and obtained a CTR of 30 ± 2 ps when the top 3% events in amplitude were selected, compared to 42 ps using all collected events. Despite their high performance, these devices presented a source of spurious coincidences originating from direct gamma-ray interactions with the MCP-PMT. In addition to deteriorating overall performance, these interactions lead to pronounced side peaks in the time-delay distribution, which complicates the analysis [78]. Ota et al. [79] presented the LFMCP-PMT, a newer version of the same photodetector without lead, that reduced that issue considerably and yielded CTRs of 35.4 ± 0.4 ps and 28.7 ± 3.0 ps without and with pulse height gating, respectively. The LFMCP-PMT is arguably the most competitive device developed to date to detect very few optical photons with very high time accuracy.

Kwon et al. [80] used the LFMCP-PMT to obtain the first cross sectional image from a positron-emitting phantom. To further enhance the time accuracy, the CNN-based method to correct for leading-edge time-walk developed by Berg and Cherry [81] was used to obtain average CTRs of 32 ± 4 ps. This study is the first that directly locates the annihilation of the positron and the electron within each LOR without a reconstruction algorithm. This study showed that sole Cherenkov emitters can provide high-SNR images with their time resolution and an accurate 3-D impact estimation.

### B. Measurements With Analog SiPMs

The first CTRs with PbF_2_ crystals coupled to SiPMs were >400 ps FWHM [39]. The same group improved these results to 297 ps FWHM with 5 × 5 × 15 mm^3^ crystals by improving the optical coupling between the crystal and the SiPMs [82]. Selecting events with single-cell amplitude, and thus removing those with optical cross-talk or after-pulse counts, improved the result to 190 ps FWHM. Kratochwil et al. [30] measured 142 and 215 ps FWHM with 2 × 2 × 3 mm^3^ and 2 × 2 × 20 mm^3^ crystals, respectively, using a low-noise high-frequency readout and applying a TWC.

It is worth noting that low-noise high-frequency readout electronics show power consumptions that prevent them to be used in systems with thousands of channels. Despite efforts to reduce the consumption in such high-performance electronics [83], [84], their use in large systems remains a big challenge. Mariscal-Castilla et al. [85] reports measurements of sole Cherenkov emitters coupled to SiPMs read out by an application-specific integrated circuit (ASIC). CTRs of >350 ps FWHM were obtained for a 3 × 3 × 5 mm^3^ TlCl crystal operated in coincidence with a fast scintillator crystal. The compromised state of the surface of the crystal measured did likely affect the measurements.

### C. PRV in Proton Beams

PGI is one of the most promising methods for PRV in PR [7], [8]. PGI uses the prompt-gammas emitted along the track of the protons to localize the Bragg peak. Prompt-gammas have energies ranging between 1 and 7 MeV, very different from the 511 keV of PET, and thus pose different constraints.

PGT uses the difference in the average time-of-flight of the protons and prompt-gammas to estimate shifts of the Bragg peak in the direction of the beam [86]. The detection time of the gamma is measured with respect to a reference signal. The first PGT measurements used the radiofrequency (RF) signal of the cyclotron [87], [88], but its shifts added jitter to the measurement [89]. Using a reference detector right at the beam nozzle has proved more effective in determining the start of the TOF [90], [91].

Rebolo et al. [47] used GATE simulations to estimate that between 80 and 150 Cherenkov photons can be detected in sole Cherenkov emitters with 10 × 10 × 10 mm^3^ with one face entirely coupled to an SiPM. Differences in this number depended on the initial gamma energy, which ranged between 2.3 and 6.1 MeV, and material choice: TlCl, TlBr, and PbF_2_. These values accounted for a realistic photon detection efficiency of the SiPMs (HPK S14160-3050HS), the extraction loss due to mismatch of index of refraction between the material and the photodetector, and losses through the crystal faces not coupled to the photodetector.

**Fig. 9.**
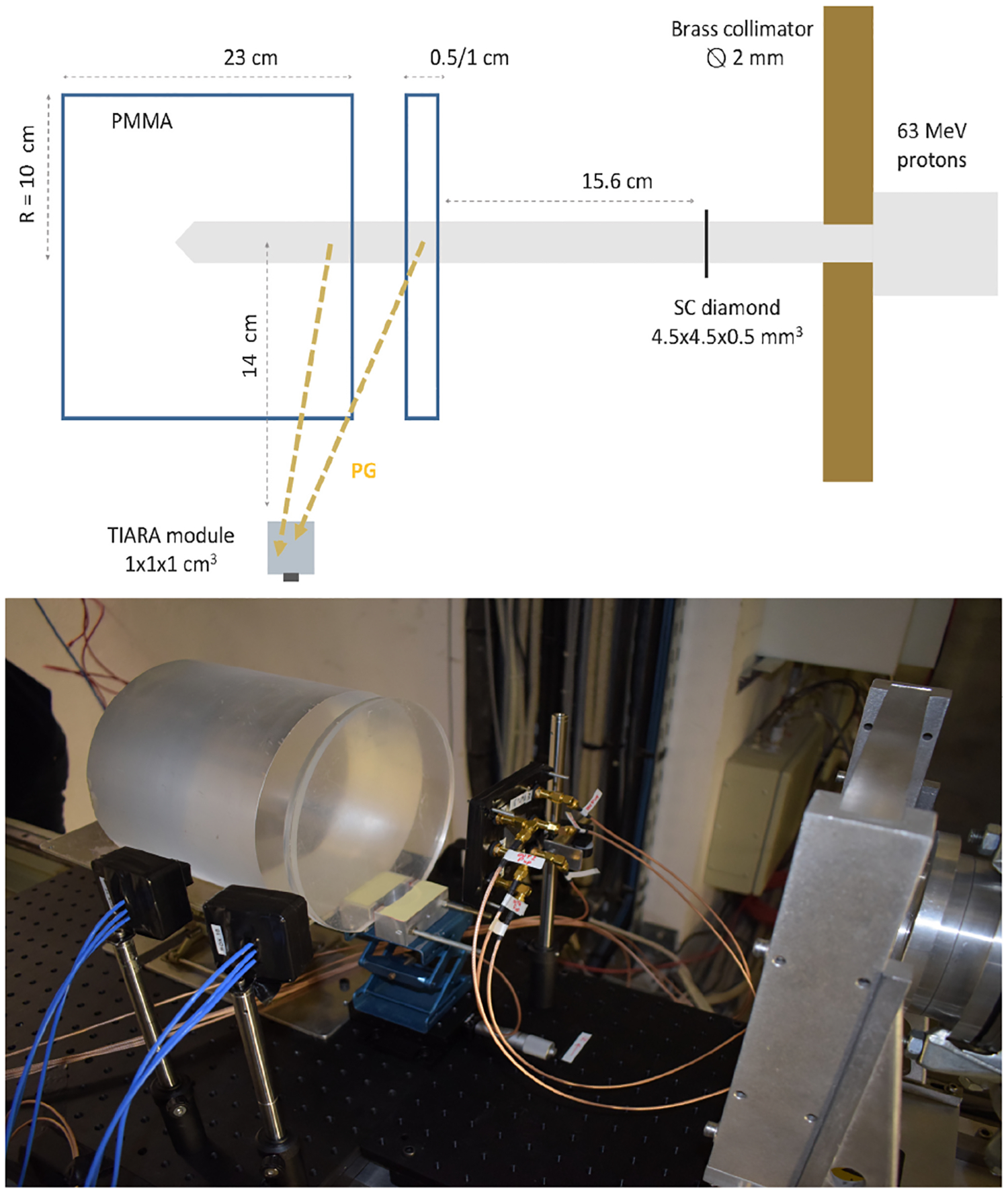
Top) illustration of the experiment in the PGTI project reported in [92]. Bottom) Photograph of the acquisition setup used in the same work. Courtesy of Dr. Sara Marcatili.

Jacquet et al. [92] reported the first PGT measurements with a sole Cherenkov emitter used as a gamma detector (Fig. 9). In this study, the prompt-gamma detection time was obtained with PbF_2_ crystals coupled to different SiPMs, while the reference detector was a diamond-based device. Two different beams were tuned to deliver approximately a single proton per proton bunch, thus referred to as single proton regime. PbF_2_ crystals of 10 × 10 × 10 mm^3^ showed a time resolution of 276 ps FWHM when operated in coincidence with a diamond detector and exposed to a 63 MeV proton beam. A similar setup with 10 × 10 × 20 mm^3^ PbF_2_ crystals and exposed to a beam with 148 MeV protons improved the time resolution to 167 ps FWHM. Such detector resolutions yielded in both cases a proton range sensitivity of 4 mm in 2*σ*.

Ellin et al. [93] used a similar approach with TlBr and TlCl crystals. In this study, the reference detector consisted of a fast plastic scintillator coupled to a photomiultiplier tube (PMT) and three different gamma detectors made of TlCl and TlBr and a beam current equivalent to 100–200 protons per bunch. Although the time resolution between the two detectors could not be disentangled due to the uncertainty added by the proton bunch width (> 2 ns), the time resolution of the gamma detectors was estimated to be between 300 and 400 ps based on prior studies. This experiment showed time uncertainties equivalent to Bragg peak shifts of 2–5 mm *σ* depending on the number of events in the distribution.

The same beam conditions and gamma detectors were used by Heller et al. [94] to quantify the improvement of the Bragg peak shift estimation when using low gain avalanche detectors (LGADs) [95] as the time reference to improve the resolution down to 1 mm. This approach, however, is heavily dependent on the statistics and an important part of its feasibility relies on the capacity to acquire events at high count rates.

A time-independent Cherenkov-based detector for PRV has been recently proposed by Rebolo et al. [96]. This design consists of a monolithic PbF_2_ crystal with dimensions of 24 × 24 × 10 mm^3^ coupled to an array of SiPMs. Position resolutions of 4–5 mm for a 1 mm slit were reported using exclusively Cherenkov light.

## V. Charge Induction and Cherenkov Emitters

Semiconductor detectors have been studied for PET systems [97], [98], [99], [100] based on their excellent energy resolution and fine 3-D segmentation [101]. Such attributes can be used to reject scatter events effectively [102] or to mitigate parallax error [103]. However, the time resolution of such high-Z gamma detectors is within 6 and 12 ns at best [104], [105], [106], far from the range of TOF-PET systems.

Measuring a CTR of <400 ps FWHM using the Cherenkov light emission in TlBr [107] enabled the possibility of using semiconductor materials for gamma applications that require a sub-nanosecond time response. Moreover, simultaneous readout of the Cherenkov and charge induction (CCI) could combine the competitive timing performance of Cherenkov light with the excellent (1%) energy resolution of TlBr at 662 keV [108], as well as the mm-like 3-D segmentation [109].

**Fig. 10.**
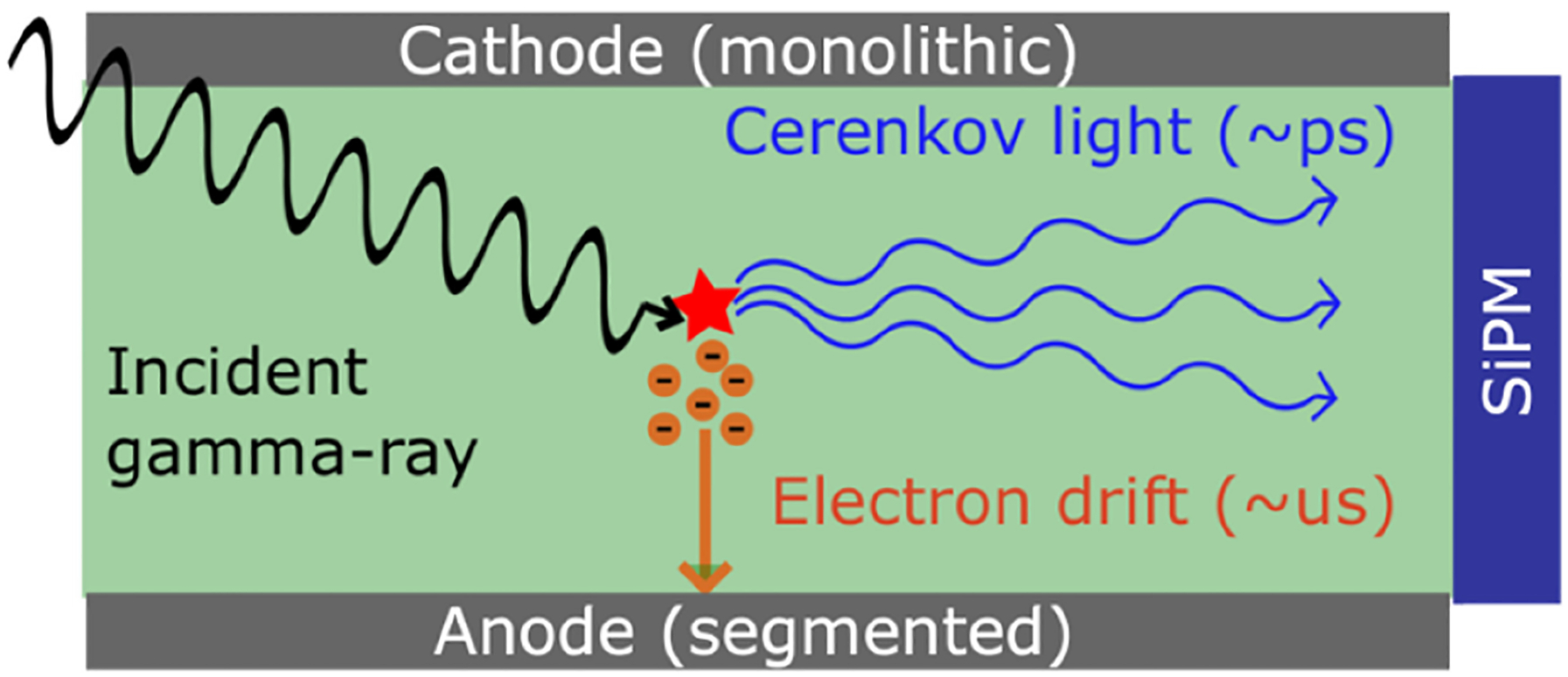
Illustration of the CCI detector concept in an electron-collecting configuration.

Ariño-Estrada et al. [110] presented the first Cherenkov CCI detector that used simultaneously the CCI and Cherenkov readout (Fig. 10).

In this study, the CTR of <400 ps obtained with a TlBr crystal against a reference detector was maintained while the bias between the electrodes was applied. A following study with a pixelated version of the CCI TlBr detector [111] showed the combination of Cherenkov and pixel anode signals in a CCI TlBr detector could provide a robust estimate of the electron drift time in the crystal, which is a very reliable estimator of the DOI position.

TlCl is a semiconductor material with physical properties comparable to those of TlBr. A CTR of 330 ps was measured using TlCl in coincidence with a reference detector and selecting events exceeding seven detected photons. The improvement over TlBr is attributed to a greater Cherenkov yield, approximately twice greater [40]. Hitomi et al. [112] reported on the time behavior of ternary TlBr_*x*_Cl_1−*x*_ compounds with different concentration of Cl^−^ with the aim of increasing the Cherenkov yield. Compounds with 50% and 70% of Cl improved the CTR measurements of the TlBr sample by approximately 15% and 30%, respectively. The only reported study on the charge properties of TlBr_*x*_Cl_(_1−*x*) show they deteriorate as a greater fraction of bromine is replaced with chlorine [113] and suggests that 50% bromine is needed to obtain a photo-peak at 662 keV.

The perovskites CsPbCl_3_ and CsPbBr_3_ [114] are attractive materials for the CCI detector concept. Similar coincidence experiments reported time resolutions of 248±8 ps and 440±31 ps FWHM, respectively [115]. The same study reported, for comparison purposes, a time resolution of 343±16 ps for TlBr. Kratochwil et al. [116] improved the CTR down to 419 ps FWHM (297 ps DTR) by measuring two 5 mm thick CsPbBr_3_ crystals in coincidence and selecting on more than 2 fired SPADs. The study also points toward an additional light component with more photons experimentally measured than Cherenkov photons simulated above 550 nm.

The first CCI detector made of CsPbBr_3_ reported similar time resolutions in simultaneous operation with the CCI readout [117]. The energy resolution of CsPbBr_3_ seems to be very competitive, with values as good as 1.6% at 662 keV [114]. Considering the improvement of energy resolution with increase of energy in semiconductor materials with similar charge drift properties, such as TlBr [118], one could expect an energy resolution between 5% and 8% at 122 keV with such material. Such range of energy allows to identify and potentially reuse events along the Compton spectrum of the 511 keV gammas.

The CCI concept has the potential to provide very high performance detectors at the expense of increased complexity due to the combination of two separate readouts. The same concern of low detection efficiency in pure Cherenkov emitters applies for this detector concept. In this case however, events that do not register a Cherenkov signal but do detect a CCI signal can still provide substantial information and probably a similar strategy to the multi kernel readout proposed for BGO [119], [120] could be used as well.

## VI. Challenges and Opportunities

This section discusses each challenge individually and transversely across all the technologies discussed. The first five subsections focus on TOF-PET, starting with the detection time aspect, following with the DOI impact on the detector performance, which is intrinsically related to the timing performance, then continuing with the scalability from a few channels to a full system, and wrapping up with a discussion on how we can squeeze all the detection information discussed before to improve the ultimate performance of TOF-PET systems. The last section discusses on the advancements with Cherenkov-based gamma detectors with application in PRV.

### A. Time Performance for TOF-PET

The photodetector intrinsic time resolution is one of the key aspects in exploiting the prompt features of Cherenkov light for TOF-PET. The development of fast photodetectors has been leading the improvements of CTR measurements. MCP-PMTs have so far shown the best results (Section IV-A), with TTS of 25 ps FWHM. However, the scalability of such devices seems more challenging than for SiPMs.

On the other hand, the most recent developments with SiPMs show SPTR values down to 28 ps FWHM (Section III-E). Such performance might be attained at a system level with high-frequency low-noise electronics with reasonable power consumption and efficient digitization and data management.

### B. Impact of DOI

An additional challenge in achieving ultrafast timing is the crystal thickness. The intrinsic time jitter caused by light transport in crystals with ≈ 10 mm thickness or greater (Section II-B) is comparable to the best CTR results achieved. Simultaneous Cherenkov and scintillation emitters with dual-ended readout (Section III-D) are likely the best positioned to provide a balance between CTR, DOI, and energy resolutions.

DOI-capable solutions with fast timing based on single-ended couplings combined with light sharing strategies in bright crystals or DOI estimation from signal time distribution have shown promise [32], [121], [122], [123] but no study has appeared to date with BGO or similar materials. CCI detectors (Section V) have a high potential to provide competitive CTR, energy and DOI resolutions, but their stage of development is significantly less mature than any of the other detector concepts discussed.

### C. Potential for System Integration

With current DOI-based time corrections, the bottleneck created by the crystal thickness seems harder to surpass than that of the detection and signal processing combined. Stacking layers of thin crystals with independent readouts using the state-of-the-art technology seems the most effective approach to achieve ultrafast timing at a system level, although at the expense of an increase in system complexity. High performance photodetector developments for broader biophotonics applications [124] and radiation detector instrumentation [125] might be of interest to TOF-PET.

Careful assembly must be considered because index mismatch between layers will decrease the collection efficiency of Cherenkov photons. In designs with high segmentation the aspect ratio is likely to increase and the optical interface between photodetectors and Cherenkov emitters (Section IV-A) would likely gain yet more relevance.

Another challenge with high segmentation approaches is the increase of intercrystal scatter events, which should be accounted for at the system level to leverage Cherenkov photons for TOF PET.

An increase in number of channels also requires developing adequate electronics circuitry, for instance, using front-end readout electronics discussed in Section III-B. Krake et al. [84], Cates and Choong [83], Latella et al. [126] report on updated circuits with less power-hungry RF amplifiers while maintaining the steep slew rate of the signal. The power consumption per channel could be reduced from ≈300 mW per channel down to 17 and 5 mW, respectively. While optimum (high) power dissipation shows CTR values for 20 mm thick BGO crystals of 271 ps FWHM, at 5 mW the values are only marginally (289 ps FWHM) worse [83]. Reduction of the PCB footprint and custom SiPM arrays led to the first 16-channel high frequency readout electronics by the same groups [61], [127] while maintaining the good single-channel performance.

In Krake and Pourashraf et al. [84], [128] a further modified circuit without balun transformer is presented, which makes design insensitive to magnetic field and is important when PET is combined with MRI.

ASICs are another approach in the direction toward a full system. They offer the advantage of low cost, small footprint and low power consumption. Piller et al. [62] and Pinto et al. [129] reported the first sub-500 ps CTR measurement with 20 mm thick BGO crystals using the FastIC ASIC, while 310 ps FWHM were obtained with thin BGO crystals. These CTRs compare to 480 ps FWHM achieved with the TOFPET2 ASIC and thin BGO crystals [61].

### D. Cherenkov-Based Proposed Systems

While no TOF-PET systems using Cherenkov-based gamma detectors are available to date, there have been proposed systems via simulation studies.

Somlai-Schweiger and Ziegler [130] simulated the performance of a system based on PbWO_4_ cubes (CHERENCUBE) coupled to position-sensitive photodetectors. Detection efficiency was identified as a major challenge of this approach, provided that the performance of the time resolution and the potential for DOI estimation of this detector deteriorated with its volume.

In Alokhina et al. [131] the simulation of a system consisting of PbF_2_ crystals with one face coupled to PMTs showed reasonable detection efficiency (130 kcps NECR at 28 kBq/cm^3^) with 10 mm thick crystals. Consuegra et al. [132] also simulated a TOF-PET system consisting of PbF_2_ crystals coupled to SiPMs. In this case, a 3-layer stack of PbF_2_ crystals with 5 mm thickness coupled to SiPMs yielded a time jitter of 29 ps FWHM without considering the contribution of the SiPM. Adding a 100 ps time spread of a hypothetical SiPM raised the total time performance to 164 ps FWHM.

Finally, Ishikawa et al. [133] proposed a dual panel system consisting of MCP-PMT photodetectors coupled to BGO. This study suggests a CTR below 40 ps FWHM can compensate for the lack of a ring-shaped geometry and obtain images with similar quality.

### E. Benefiting From Cherenkov Photons in TOF-PET

Eventually, the performance gain with prompt photon-based detectors needs to be translated to full systems, where harnessing the timing and unique information provided by Cherenkov photons remains complex. Large-scale high-fidelity studies that capture detailed timing information from optical photons can provide insight on how to utilize these photons. Though a complex task, it certainly mobilizes the PET community through dedicated taskforces, scientific challenges [1], and workshops (e.g. Fast Timing in Medical Imaging workshop, June 2022) [4].

Specifically, recording individual event time stamps and optical triggers to build “mixed” coincidences between detectors triggered on “prompt” or “slow” photons in a full ring is an unsolved challenge. Such detailed information on the nature of TOF kernels at the system level remains limited, albeit necessary [2], [119]. The TOF kernels have a complex shape defined by the nature of the coincidence (prompt-prompt, mixed prompt-slow, slow-slow) and become event-dependent. Applying a single Gaussian kernel to all events is no longer valid when coincidences are formed by different emission mechanisms. TOF kernels in the reconstruction contribute to the SNR [120] and thus need to be evaluated carefully to translate the benefits of prompt photon detectors to a full ring, which may be the ultimate challenge.

### F. Strong Light Mechanism for PRV

Leveraging Cherenkov light emission in PRV is a much less challenging task than it is for TOF-PET thanks to the much greater light yield brought by high energy prompt-gammas. Demands are also different. Despite the need for fast timing to obtain time-of-flight measurements of the proton plus the prompt-gamma in PGT, time resolution is often limited by the proton bunch width, which is often a few hundred ps. An important challenge in PGI is the time-structure of the beams, which tend to concentrate the emission of prompt-gammas in very narrow windows of time. The complication here is twofold: in the first place because count rates are very high. The second is that, despite the high-count rates, the total available events within these windows can be scarce. And, in addition, the radiation background conformed by fast neutrons and 511 keV gammas is very aggressive.

Cherenkov light, when compared to scintillation light, offers several important benefits: it allows for fast count rates because of the quick emission of Cherenkov light, always within the ps scale, and allows to reject background more efficiently. The main drawback of Cherenkov-based detectors in this application is the lack of fine spectroscopic resolution.

However, preliminary data discussed in Section IV-C hints the aforementioned features of such detectors can compensate for the poor energy resolution in either PGT or collimation-based imaging methods for PRV. Additionally, the CCI signal in CCI detectors (Section V) could provide such spectroscopic performance.

## VII. Conclusion

Cherenkov light is one of the most promising physics-based advances in the world of nuclear imaging and therapy, whether it is to enable a leap in image quality or advance therapy through accurate, patient-specific radiation delivery monitoring. Many of the technical and computational shortcomings described above are likely to be overcome and lead to new ways of performing imaging. These shortcomings might be addressed with a combination of material science development and testing, hardware improvements, as well as algorithmic developments to harness the unique signature of Cherenkov light.

## Figures and Tables

**TABLE I:** Physical Properties and Calculated Cherenkov Yield (N_Ckv_) Based On the Frank–Tamm Equation of Various Materials That Can Serve as Potential Cherenkov-Based Detectors

Material	Chemical^a^ composition	Density [g/cm^3^]	Z_*eff*_^b^	*τ*_att_/*τ*_att,p_^c^ [mm]	Melting point [°C]	n^d^	LY^e^ [ph/keV]	*τ*_*d*,*eff*_^f^ [ns]	Peak λ [nm]	Cutoff λ [nm]	N_Ckv_^g^ [ph]	References
BaF_2_	BaF_2_	4.9	51	21.4 / 111	1368	1.48	8.5	3.7	220	180	25.1	[26], [134], [135]
BGO	Bi_4_Ge_3_O_12_	7.1	71	10.1 / 24.1	1050	2.12	10.7	230	480	310	18.3	[26], [136]
BSO	Bi_4_Si_3_O_12_	6.8	74	9.8 / 21.5	1025	2.06	2.0	59	480	290	19.8	[68], [69]
CsPbBr_3_	CsPbBr_3_	4.8	62	19.2 / 56.8	567	2.40	/	/	1/λ^2^	555	9.3	[115], [137]
CsPbCl_3_	CsPbCl_3_	4.2	66	20.3 / 52.8	480	1.91	/	/	1/λ^2^	435	13.8	[115], [138]
GAGG:Ce(Mg)	Gd_3_Al_2_Ga_2_O_12_	6.6	51	15.8 / 77.6	1850	1.87	40	80	540	490^j^	9.3	[139], [140]
LaBr_3_:Ce	LaBr_3_	5.1	45	22.0 / 161	1116	2.1	63	25	380	340	25.6	[141], [142]
LSO:Ce(Ca)	Lu_2_SiO_5_	7.4	64	11.2/33.8	2050	1.81	42	40	420	370	10.1	[23], [143]–[145]
LuAG:Ce	LU_3_Al_5_O_12_	6.7	59	11.1 / 46.2	2020	1.84	25	70	535	470^h^	14.9	[146]–[149]
LuAG:Pr	LU_3_Al_5_O_12_	6.7	59	11.1 / 46.2	2043	1.84	15	20	320	300	15.7	[147], [149], [150]
LYSO:Ce(Ca,Mg)	Lu_2(1−*x*)_ Y_2*x*_SiO_5_	7.0	62	11.9/35.8	2050	1.81	42	40	420	370	10.6	[23], [144], [145], [151]
NaI:Tl	NaI	3.7	50	28.7 / 162	651	1.78	38	239	415	250	44.3	[152], [153]
NE111/BC422/EJ232	C_9_H_10_	1.0	6	103 / ≫1000	70	1.58	10.1	1.5	370	350	15.4^i^	[26], [154]
PbF_2_	PbF_2_	7.8	77	8.6 / 18.5	824	1.77	/	/	1/λ^2^	250	17.9	[30], [155]
PWO	PbWO_4_	8.3	74	8.5 / 19.6	1123	2.26	0.02	10	420	340	15.3	[156]–[159]
TlBr	TlBr	7.5	73	9.7 / 22.8	460	2.47	/	/	1/λ^2^	440	12.0	[107], [160]
TlCl:Be,I	TlCl	7.0	76	9.7 / 21.1	430	2.28	0.9	60	430	395	14.4	[28], [40], [161], [162]
YAG:Ce	Y_3_Al_5_O_12_	4.6	29	25.5 / 677	1970	1.84	30	70	550	490^k^	15.3	[163], [164]

a Chemical composition does not include (co-)dopance material.

b The effective atomic number is energy dependent and depends on the type of interaction. It is approximated according to [165] with an exponent of 2.94 and rounded to the next integer.

c Gamma-ray attenuation length calculated according to NIST database at 500 keV, considering all interactions. τ_att,p_ considers only photoelectric interactions.

d When available, the refractive index *n* is extracted from [166] at 550 nm.

e The number of produced scintillation photons per unit of energy deposition depends on the exact composition, (co-)doping, and varies from different producers.

f Effective decay time calculated as the harmonic average of the individual decay time components according to [26]. The scintillation decay time can vary based on the composition of (co-)dopance and different producers.

g Number of produced Cherenkov photons following a 511 keV gamma-ray photoelectric interaction, between the cutoff wavelength and 900 nm. Calculations based on the material properties according to [28].

h Depending on the doping concentration, also transparent between 270–330 nm and 360–420 nm.

i For Compton interaction with a recoil energy of 341 keV and a total electron track length of about 1 mm. For an electron with 511 keV the Cherenkov yield increases to ≈ 43 photons, with an electron track length of about 2 mm.

j Depending on the doping concentration, also transparent between 360–400 nm.

k Depending on the doping concentration, also transparent between 350–420 nm.
